# 유방암 생존자의 생활습관, 우울, 부부친밀도가 삶의 질에 미치는 영향

**DOI:** 10.4069/kjwhn.2020.03.05

**Published:** 2020-03-18

**Authors:** Su-Jin Seo, Ju-Hee Nho, Myoungha Lee, Youngsam Park

**Affiliations:** 1 Department of Nursing, Kunjang University College, Gunsan, Korea; 1 군장대학교 간호학과; 2 College of Nursing, Jeonbuk Research Institute of Nursing Science, Jeonbuk National University, Jeonju, Korea; 2 전북대학교 간호대학•간호과학연구소; 3 Department of General Surgery, Presbyterian Medical Center, Jeonju, Korea; 3 전주예수병원 외과

**Keywords:** Life style, Depression, Marriage relationship, Quality of life, Breast neoplsms, 생활습관, 우울, 부부친밀도, 삶의 질, 유방암

## Abstract

**Purpose:**

This study investigated lifestyle, depression, marital intimacy, and quality of life (QoL) in breast cancer survivors, with the goal of identifying the impacts of these factors on QoL.

**Methods:**

A sample of 146 breast cancer survivors was surveyed in this cross-sectional study. Data were collected from March 20 to May 30, 2019, using self-report structured questionnaires at a hospital located in Jeonju, Korea. Data were analyzed using the independent t-test, analysis of variance, Pearson correlation coefficients, and hierarchical regression analysis. Participants agreed to complete a face-to-face interview, including administration of the Health Promoting Lifestyle Profile II, Depression Anxiety Stress Scale 21-Depression Scale, Marital Intimacy Scale, and Functional Assessment Cancer Therapy-Breast Cancer tool.

**Results:**

QoL was positively correlated with lifestyle (r=.49, *p*<.001) and marital intimacy (r=.45, *p*<.001) and negatively correlated with depression (r=–.72, *p*<.001). Hierarchical multiple regression analysis showed that depression (β=–0.63, *p*<.001), marital intimacy (β=0.19, *p*=.001), and lifestyle (β=0.13, *p*=.031) had significant effects on the QoL of breast cancer survivors, accounting for 63.3% of variance in related QoL.

**Conclusion:**

This study provides insights into how breast cancer survivors’ QoL was influenced by depression, marital intimacy, and lifestyle. To improve the QoL of breast cancer survivors, healthcare providers should consider developing strategies to decrease depression, to increase marital intimacy, and to improve lifestyle.

## Introduction

### 연구 필요성

우리나라 유방암은 발병연령이 낮고 30–50대에서 많이 발병하고 있어 장기간 생존자로 살아가는 생존연령이 길어지고 있다[1]. 암 생존자란 암 진단을 받고 관련 치료가 끝난 후 생존해 있는 사람으로, 2017년 국가암등록 통계에 따르면 유방암 발생률은 10만 명당 43.7%이고 암 생존자는 100만 명에 이르며 유방암 5년 상대생존율은 93.2%이다[2]. 이는 암 유병자가 계속적으로 증가하는 추세로 이제는 암 치료와 함께 암 생존자의 건강 증진과 재발 예방에 관심을 가져야 할 필요성이 증가하고 있음을 나타낸다[3].

유방암 발생 원인을 살펴보면 비만, 흡연, 음주, 식이 등의 생활습관이 주요한 것으로 나타나, 건강한 식이, 체중 조절, 신체활동, 금연과 금주 등의 건강한 생활습관이 유방암을 34%까지 감소시킬 수 있다고 제시하였다[4,5]. 이렇게 유방암 발생과 생활습관이 밀접한 관련이 있으므로 올바른 생활습관이 건강한 삶의 결과라고 할 수 있다. 치명적인 말기 질환으로 인식되었던 유방암은 이제 그 생존율의 증가에 따라 만성질환으로 재인식되고 있다. 이에 따라 유방암 생존자의 건강 관리를 위한 생활습관의 중요성이 제시되는 바, 유방암 환자가 포함된 암 생존자의 건강행위에 대한 연구 결과 암 진단 이후 건강행위 실천율이 시간이 지남에 따라 감소되어 유방암 생존자의 생활습관이 완전히 개선되기 어려움을 알 수 있다[6]. 따라서 유방암 생존자들에 대한 생활습관을 파악하여 건강한 생활습관을 이행할 수 있는 전략을 개발함으로써 삶의 질 향상을 기대해 볼 수 있다.

유방암 생존자는 암을 진단받고 희망 없음, 신경질적임, 가치 없음, 불안, 우울, 절망 등 부정적인 감정을 겪으며 불유쾌한 정서적 경험을 하게 된다[7]. 이렇게 유방암 환자들이 암 진단 시에 겪는 심리적 디스트레스(distress) 중 우울은 가장 높게 측정이 되며 생존 시기 동안 지속된다고 보고되었다[8]. 미국의 유방암 생존자 250명을 대상으로 한 연구에서[9] 유방암 생존자의 우울은 일반인의 우울보다 더 빈도가 높고 인지적, 정서적, 역할적, 신체적, 사회적 기능의 삶의 질이 모두 낮게 나타나 삶의 질에 주요한 영향요인임을 제시하였다. 더욱이 유방암 생존자들은 일차 치료가 종료된 후에 피로, 어깨운동 장애, 신체 통증 등의 신체적 증상, 우울, 불안, 성적 욕구 감소 등의 심리적 문제, 직업 유지와 자녀 양육 등의 사회적 측면에서 취약한 상태가 되므로[10,11] 이에 따른 건강문제의 해결이 필요하며 더 나아가 삶의 질 개선에도 전략이 필요하다[3].

유방암 진단은 환자뿐 아니라 배우자에게도 심각한 충격으로 받아들여진다. 유방암 생존자의 43.9%가 유방암 진단을 암으로 인해 위기에 처했다고 인지하거나 높은 수준의 스트레스로 인지하는 외상으로 받아들이며, 배우자 또한 24.6%가 외상으로 인지한 결과가 보고되었다[12]. 치료과정인 유방절제술, 항암화학요법, 방사선 요법과 호르몬 요법 등으로 여성성의 상실감을 경험하며 생리가 중단되고 탈모로 인해 신체적인 변화도 더 심해지며 불안감이 유발되고 부부관계까지 위축되기도 한다[10]. 긍정적인 부부친밀도는 유방암 환자의 심리적 사회 적응을 증진시키고[13], 배우자는 환자에게 가장 가까운 지지체계로 암 환자의 회복, 위기 대처, 변화 적응에도 중요한 요소이다. 유방암 환자에서 배우자와의 긍정적인 관계는 우울을 감소시키고 질병 대처능력을 키워 건강이 증진되며 더 나아가 삶의 질까지도 향상하게 된다[14].

유방암을 치료 중인 환자의 생활습관, 우울, 부부친밀도는 삶의 질과 건강에도 영향을 미친다[5,9,14]. 그러나 유방암 생존자의 생활습관, 우울, 부부친밀도를 확인하고 이것이 삶의 질에 미치는 영향을 파악한 연구는 미흡한 실정으로 유방암 생존자 대상으로는 신체적인 문제, 심리적 문제와 부부친밀도 등을 각각 확인한 연구가 대부분이었다[10-12,14]. 이에 본 연구에서는 유방암 생존자의 생활습관, 우울, 부부친밀도와 각각 변수들 간의 관계를 파악하고 유방암 생존자의 삶의 질에 영향을 미치는 변수들을 통합적으로 확인하여, 유방암 생존자의 삶의 질을 향상시키기 위한 효과적인 간호중재 프로그램 개발에 필요한 자료를 제공하고자 한다.

### 연구 목적

본 연구의 목적은 유방암 생존자의 생활습관, 우울, 부부친밀도와 삶의 질을 확인하고 이러한 요소가 삶의 질에 영향을 미치는 영향을 파악하기 위한 연구로, 구체적인 목적은 다음과 같다.

1) 유방암 생존자의 생활습관, 우울, 부부친밀도, 삶의 질을 파악한다.

2) 유방암 생존자의 특성에 따른 삶의 질을 파악한다.

3) 유방암 생존자의 생활습관, 우울, 부부친밀도, 삶의 질 간의 상관관계를 파악한다.

4) 유방암 생존자의 생활습관, 우울, 부부친밀도가 삶의 질에 영향을 미치는 요인을 파악한다.

## Methods

Ethics statement: This study was approved by the Institutional Review Board (IRB) of Presbyterian Medical Center Hospital (IRB no: 2019-03-007). Informed consent was obtained from the subjects

### 연구 설계

본 연구는 유방암 생존자의 생활습관, 우울, 부부친밀도가 삶의 질에 영향을 미치는 영향을 확인하기 위한 서술적 조사연구이다.

### 연구대상 및 표집방법

본 연구는 전주에 소재한 종합병원에서 유방암 진단을 받고 수술, 항암화학요법, 방사선 요법 및 호르몬 요법 치료가 끝난 후 추적 관리를 위해 외래로 내원한 생존자를 대상으로 하였다. 연구 참여 선정 기준은 연구 참여에 동의한 만 20세 이상의 배우자가 있는 기혼 여성을 대상으로 하였다. 재발했거나 현재 치료 중인 환자, 심각한 정신질환을 진단받고 치료 중인 자, 심장혈관 질환, 호흡기계 질환, 합병증이 있는 고혈압이나 당뇨, 만성 신부전 등 유방암 이외 다른 급, 만성 질환을 가지고 있는 자는 제외하였다. 연구 대상자수는 G*Power 3.1 프리웨어를 사용하였으며, 다중회귀분석을 시행하였는데 유의수준 .05, 효과 크기 .15, 검정력 .85, 예측변수 13개를 투입했을 때 최소한의 표본의 수는 144명으로 산정되었다. 이에 180명의 연구 대상자에게 설문지를 배부한 후 160부를 회수하였다. 이 중 응답이 완전하지 않거나 불성실한 설문지 14부를 한하고 146부를 최종 자료 분석에 사용하였다.

### 연구 도구

본 연구에 사용한 도구는 생활습관, 우울, 부부친밀도, 삶의 질 도구이다. 이들 측정도구는 이메일을 통해서 연구자가 직접 측정도구의 개발자에게 도구 사용에 대한 동의를 받고 사용하였다.

#### 생활습관

본 연구에서는 Walker 등[15]이 개발한 Health Promoting Lifestyle Profile II (HPLP II)를 한국어로 번안한 도구[16]를 사용하였다. HPLP ¬Ⅱ는 각각 6개 영역에 걸쳐서 총 52문항으로, 건강에 대한 책임감(9문항), 영양(9문항), 운동(8문항), 영적 성장(9문항), 대인관계(9문항), 스트레스 관리(8문항)으로 구성되어 있다. 생활습관 행위 이행 정도에 따라 ‘전혀 하지 않는다’ 1점, ‘가끔 한다’ 2점, ‘자주 한다’ 3점, ‘규칙적으로 한다’ 4점의 Likert 척도로, 점수가 높을수록 생활습관 행위 이행 정도가 높은 것을 의미한다. Walker 등[15]이 개발했을 당시 Cronbach’s ⍺=.93이었고[15], 본 연구에서는 Cronbach’s ⍺=.94였다.

#### 우울

유방암 생존자의 우울은 Henry와 Crawford [17]가 개발한 우울, 불안, 스트레스 척도(Depression Anxiety Stress Scale)중 연구용으로 개방된 한국어판 우울측정도구를 사용하였다. 우울측정도구는 총 7문항으로 되어 있고 낮은 긍정감, 낮은 자존감 및 의욕, 절망감으로 구성되어 있다. ‘나에게 전혀 적용되지 않는다’ 0점, ‘나에게 매우 많이 또는 대부분 적용된다’ 3점의 Likert 4점 척도로 총 점수는 0점에서 21점까지이며 점수가 높을수록 우울이 심함을 나타낸다. 우울점수가 10점 이상일 경우 증상이 있음을 의미한다[17]. 개발 당시 Cronbach’s ⍺=.88이었고[17] 본 연구에서는 Cronbach’s ⍺=.96이었다.

#### 부부친밀도

본 연구에서는 Lee [18]가 개발한 부부친밀감 도구를 사용하였다. 인지 영역 5문항, 정서 영역 5문항, 성 영역 5문항의 총 15문항으로 이루어져 있으며, 각 문항은 ‘전혀 그렇지 않다’ 1점에서 ‘매우 그렇다’ 5점까지 Likert 5점 척도이다. 점수가 높을수록 부부친밀도가 높음을 의미한다. 본 도구는 개발 당시 Cronbach’s ⍺=.90이었고[18] 본 연구에서는 Cronbach’s ⍺=.90이었다.

#### 삶의 질

유방암 생존자의 삶의 질은 Functional Assessment Cancer Therapy-Breast Cancer (FACT-B)의 한국어판 도구[19]를 사용하였다. FACT-B는 5개 영역 총 37문항으로, 신체적 상태(7개 문항), 사회적/가족 상태(7개 문항), 정서적 상태(6개 문항), 기능적 상태(7개 문항), 유방암 특이적 상태(10개 문항)로 구성되어 있다. 증상이 없는 경우에 0점, ‘조금 그렇다’로 지각한 경우에 1점에서부터 ‘매우 그렇다’에 4점을 부여하는 5점 Likert 척도로서 점수가 높을수록 삶의 질이 높음을 의미한다. 국내 연구에서는Cronbach’s ⍺=.90이었고[19], 본 연구에서는 Cronbach’s ⍺=.92였다.

### 자료 수집

본 연구는 2019년 3월 20일부터 5월 30일까지 전주에 소재한 종합병원에서 유방암 진단을 받고 치료가 끝난 후 추후 관리를 위해 외래에 내원한 유방암 생존자들에게 연구의 목적과 취지, 연구 참여자로서의 권리, 비밀 보장을 설명하여 자발적인 의사로 연구 참여를 희망한 자에게 연구 참여 동의서를 받고 자가 보고식 설문지를 연구자가 직접 배부하였다. 자료 수집은 비교적 조용한 유방외과 회의실에서 이루어졌고, 설문지 작성에 소요되는 시간은 20–25분이었으며 응답한 설문지는 바로 회수하였다. 작성에 참여한 대상자들에게는 소정의 선물을 제공하였다.

### 자료 분석

수집된 자료는 IBM SPSS Statistics ver. 24.0 (IBM Corp., Armonk, NY, USA)을 이용하여 다음과 같이 분석하였다.

1) 유방암 생존자의 특성과 생활습관, 우울, 부부친밀도, 삶의 질에 대한 실수와 백분율, 평균과 표준편차로 산출하였다.

2) 유방암 생존자의 특성에 따른 삶의 질 차이는 independent t-test, one-way ANOVA로 분석하고 사후 분석은 Scheffé test를 이용하였다.

3) 유방암 생존자의 생활습관, 우울, 부부친밀도, 삶의 질 정도 간의 상관관계는 Pearson’s correlation coefficients를 산출하였다.

4) 유방암 생존자의 삶의 질에 대한 영향요인을 파악하기 위해 위계적 회귀분석(hierarchical regression analysis)을 실시하였다.

## Results

### 대상자의 일반적 특성과 생활습관, 우울, 부부친밀도와 삶의 질

본 연구 대상자의 나이는 평균 55.2세로 50–59세가 55명(37.7%), 교육수준은 고등학교 졸업이 67명(45.9%)으로 가장 많았다. 직업을 가지고 있지 않은 경우가 91명(62.3%), 종교가 있는 대상자가 109명(74.7%)이었고, 가족의 평균 월수입은 200만 원에서 400만 원 사이가 65명(44.5%)으로 가장 많았다. 암 진단병기는 2기가 64명(43.8%)으로 가장 많았고, 암 진단기간은 1년 이내가 61명(41.8%)이었다. 암 치료방법은 수술, 항암, 방사선 치료를 같이 한 경우가 60명(41.1%)으로 가장 많았다(Table 1). 대상자의 생활습관 점수는 평균 141.13±23.48점으로 나타났고, 우울의 평균 점수는 6.52±5.31점으로 106명(72.6%)이 우울 증상이 있었다. 부부친밀도 점수는 평균 45.47±9.95점, 삶의 질 점수는 평균 86.98±24.00점이었다(Table 2).

### 대상자의 일반적 특성에 따른 삶의 질 차이

본 연구 유방암 생존자의 삶의 질 점수는 대상자의 나이와 배우자의 나이에 따라 차이가 있었다.사후분석 결과 대상자와 배우자의 나이가 50대 이상이 50대 미만보다 점수가 더 높게 나타났다(F=7.25, *p*=.001; F=5.57, *p*=.005). 또한 직업이 있는 대상자의 삶의 질 점수가 더 높았다(t=2.34, *p*=.021) (Table 3).

### 생활습관, 우울, 부부친밀도와 삶의 질과의 관계

본 연구 대상자의 삶의 질 점수는 생활습관 점수가 높을수록 높게 나타났다(r=.49, *p*<.001). 또한 우울 점수가 낮을수록(r=–.72, *p*<.001), 부부친밀도 점수가 높을수록 삶의 질 점수가 높게 나타났다(r=.45, *p*<.001) (Table 4).

### 생활습관, 우울과 부부친밀도가 삶의 질에 미치는 영향

삶의 질 관련 요인을 확인하기 위해 위계적 회귀방법에 따라 다중회귀분석을 실시하였다. 일반적 특성 중 대상자의 나이, 배우자 나이, 직업을 영향요인에 추가하여 분석한 결과, model 1에서는 대상자 나이(β=.33, *p*=.005)와 직업(β=–.27, *p*=.001)이 삶의 질에 영향을 미쳤고, 생활습관이 추가된 model 2에서는 대상자 나이(β=.24, *p*=.032), 직업(β=–.21, *p*=.005)과 생활습관(β=.35, *p*<.001)이 영향을 미쳤다. 우울이 추가된 model 3에서는 대상자 나이(β=.18, *p*=.035), 생활습관(β=–.22, *p*<.001), 우울(β=–.65, *p*<.001)이 삶의 질에 영향을 미쳤다. 최종 회귀모형에서 우울(β=–.63, *p*<.001), 부부친밀도(β=.19, *p*=.001), 생활습관(β=.13, *p*=.031)이 삶의 질에 영향을 미치는 요인으로 나타났으며 이들 변수가 유방암 생존자의 삶의 질을 설명하는 설명력은 63.3%이다. 유방암 생존자의 삶의 질에 우울이 가장 큰 영향을 미쳤고 그 다음으로 부부친밀도, 생활습관 순이었다. 즉, 우울이 낮을수록, 부부친밀도가 높을수록, 생활습관이 좋을수록 유방암 생존자의 삶의 질이 높아지는 것으로 나타났다. 회귀 모형의 적합도는 다중공선성 검정으로 공차한계(tolerance)가 0.43–0.93으로 0.1 이상이었고, 분산팽창지수(variance inflation factor)는 1.07–2.34로 기준치 10 이하로 나타나 독립변수 간의 다중공선성의 문제는 없었다. 또한 모형의 정규성과 잔차 자기상관성을 검정한 결과 Dubin–Watson 통계량은1.600로 측정되어 잔차의 자기 상관성이 없었으며, Kolmogorov–Smirnov 결과(*p*=.456)에서도 잔차의 정규성을 만족하였기 때문에 본 회귀모형이 적합한 것으로 나타났다(Table 5).

## Discussion

본 연구는 유방암 생존자의 생활습관, 우울, 부부친밀도와 삶의 질 수준을 파악하고, 유방암 생존자의 삶의 질에 미치는 영향요인을 확인한 연구이다. 연구 결과 유방암 생존자의 삶의 질은 생활습관이 좋을수록, 우울이 낮을수록, 부부친밀도가 높을수록 높아지는 것으로 나타나, 유방암 생존자의 삶의 질을 증진시키기 위한 간호중재 프로그램 개발에 기초자료로 활용될 수 있을 것이다.

본 연구에서 유방암 생존자의 삶의 질 점수는 148점 만점 중 평균 86.98±24.00점으로 유방암 생존자를 대상으로 같은 도구를 사용하여 삶의 질을 연구한 국내외 선행연구[3,11]와 비슷한 결과였고, 대상자의 나이와 배우자의 나이가 많을수록 높은 것으로 나타났다. 이는 젊은 유방암 생존자의 삶의 질이 나이 든 생존자보다 낮다는 선행연구[20]와 일치한다. 특히, 50세 미만의 유방암 생존자가 50세 이상의 생존자보다 삶의 질이 낮게 나왔는데, 이는 폐경이 되는 시점인 51세를 기준으로 50세 이하를 ‘젊은 유방암 환자’라고 지칭하는 나이[21]와도 유사한 결과이다. 이는 발병연령이 낮아져 생존기간이 길어진 젊은 유방암 생존자가 다양한 신체, 심리, 생식, 관계, 사회적인 문제들을 더욱 많이 경험하면서 삶의 질이 낮게 나타나는 것으로 보인다. 특히 젊은 유방암 생존자는 치료와 관련된 감소된 생식기능, 어린 자녀의 양육, 젊은 나이에 심각한 증상 경험, 직업 스트레스 등과 낮은 신체상 등을 더 많이 경험한다고 하였다[9,22,23]. 따라서 유방암 생존자의 나이에 따른 삶의 질에 대한 사정뿐 아니라, 젊은 유방암 생존자가 경험하는 다양한 문제들을 확인하고 조절할 수 있는 의료진의 노력이 필요하다. 또한, 본 연구에서는 유방암 생존자 배우자의 나이에 따라서도 삶의 질에 차이가 있는 것으로 나타났다. 이는 508명의 유방암 생존자 배우자를 대상으로 삶의 질을 파악한 연구[10]에서 보고한 젊은 배우자의 삶의 질이 더 낮다는 결과와도 일치한다. 특히 젊은 유방암 생존자는 우울, 성기능과 결혼 만족도에 더 많은 어려움을 경험하는 것으로 보고되어[10], 젊은 유방암 생존자와 더불어 배우자의 심리적인 문제와 부부관계를 사정하고 이를 개선하기 위한 중재 개발이 필요할 것으로 보인다.

본 연구에서 생활습관이 좋을수록 삶의 질이 높아, 생활습관이 삶의 질에 영향을 미치는 중요한 요소로 파악되었다. 이는 유방암 생존자의 생활습관이 좋을수록 삶의 질도 높다는 연구[24]와도 맥락을 같이한다. 생활습관 하위영역 중 신체활동 영역의 점수가 제일 낮게 나타났는데, 이는 암 생존자가 실제적으로 신체활동이 제대로 이루어지지 않고 있다는 선행연구[6,25]의 결과와 유사하다. 최신 의학 발전과 적극적인 보조요법의 발달로 암 환자 생존율이 계속적으로 높아짐에 따라, 암이라는 질병을 만성질환으로 간주하고 생활습관을 건강하게 관리하는 것이 더 중요해졌다. 유방암 생존자의 신체활동량은 중년기 성인 혹은 다른 암 환자들과 유사한 수준이었으나 대부분 걷기 수준의 낮은 강도로 이루어지며 피로감, 체력 부족, 통증 등이 방해요소로 보고되었다[25]. 이러한 결과는 유방암 생존자를 대상으로 운동 관리를 포함한 지속적인 건강 관리가 필요함을 제시한다. 대부분의 암 생존자들은 병원 치료가 끝나면 정기적인 병원 방문 이외에 주기적으로 참여하는 프로그램이 많지 않다. 따라서 건강한 생활습관을 통한 관리의 중요성을 인식하고 본인 스스로 의지를 갖지 않으면 실천하기가 어렵다. 유방암 생존자의 가족 지지와 사회적 지지가 건강한 생활습관을 유지, 증진할 수 있는 요소라고 하였으므로[26], 동기 부여를 위해 지지체계를 활용한 생활습관 중재 프로그램 개발과 적용을 통해 건강한 생활습관을 이행하면 삶의 질 향상까지 이어질 수 있을 것이다.

본 연구에서 우울은 유방암 생존자의 삶의 질에 가장 큰 영향을 미치는 것으로 나타났다. 또한 우울증을 경험하고 있는 대상자가 72.6%를 차지하고 있어 대부분의 유방암 생존자들의 심리적 문제 중 대표적인 증상을 우울로 볼 수 있다. 우울의 빈도는 미국의 유방암 생존자를 대상으로 한 연구[9]에서 PHQ-8 (Patient Health Questionnaire-8) 도구를 사용하여 나타난 빈도인 16%보다 높았는데, 이는 대상자의 차이라고 생각된다. 즉, 선행연구에서는 치료 후 기간이 6년 이상 대상자로 3기 이상인 환자가 5%인 반면 본 연구는 치료 후 기간이 3개월 이상이고 3기 이상인 대상자가 43%였는데, 이로 인한 차이로 보인다. 본 연구의 결과는 유방암 생존자의 우울이 높을수록 삶의 질이 낮고, 우울이 삶의 질에 가장 큰 영향을 미치는 요인이라는 선행연구[9,22,23]와 일치된 결과이다. 유방암 생존자는 치료로 인한 신체적 변화와 함께, 우울, 불안, 수면 장애, 재발에 대한 두려움과 불안 같은 심리적 디스트레스 및 배우자와의 낮은 결혼 만족도 등의 문제들로 인해 우울을 경험한다고 하였다[9,22,23]. 따라서 유방암 생존자들에 대한 의료진의 관심은 물론 우울에 대한 사정 및 우울을 경험하는 대상자의 우울을 감소시킬 수 있는 심리적인 중재가 필요하다. 또한 사회경제적 요소는 우울뿐 아니라 삶의 질과도 관련이 있다고 보고되어 있으므로[27], 유방암 생존자들의 건강 형평성에 맞춘 사회경제적 요소를 고려한 전략을 포함한 프로그램 중재를 고려해 볼 필요가 있겠다.

본 연구에서 부부친밀도는 평균 45.47±9.95점으로 동일한 도구를 사용하여 측정한 부인암 환자의 부부친밀도 점수인 51.1점[28]보다 낮게 측정되었다. 선행연구에서 유방암 생존자는 치료로 인해 감소된 성기능으로 인한 부정적인 인식이 부부친밀도를 유의하게 감소시킨다고 한 것과 같이[29],유방암 생존자들이 다양한 치료로 인한 신체, 심리, 관계적 문제를 경험하며 부부친밀도가 감소한 것으로 보인다. 또한 긍정적인 부부친밀도는 유방암 생존자의 심리•사회적 적응과 삶의 질에 영향을 미쳐, 유방암 생존자 여성의 부부친밀도를 높이는 프로그램은 유방암 생존자 여성의 신체적, 배우자와의 관계적, 그리고 사회적 측면에서 긍정적인 효과를 나타냈으므로[14], 유방암 생존자와 배우자를 모두 포함시킨 부부친밀도 향상 프로그램을 개발하여 적용하는 것이 필요하다.

본 연구는 치료 종료 후 3개월 이상 경과한 생존자를 대상으로 하여 변수를 확인한 제한점이 있다. 유방암 생존자는 치료 후 경과기간에 따라 삶의 질에 차이가 난다고 하였으므로[11] 추후 치료 후 경과기간별로 구분한 유방암 생존자의 삶의 질 영향요인을 파악하는 연구를 제언한다. 또한 본 연구에서는 수술의 종류와 방법을 구별하지 않고 수술 여부로만 확인하였는데, 수술의 형태 또는 유방재건술 등이 우울이나 삶의 질에 더 큰 영향을 줄 수 있다는 제한점이 있다. 본 연구에서는 유방암 생존자 여성만을 대상으로 하였으므로 추후 유방암 부부를 대상으로 변수들을 파악하여 차이를 확인해보는 연구를 제언한다. 이러한 제한점에도 불구하고 본 연구는 유방암 생존자를 대상으로 신체, 심리, 관계적인 측면을 나타내는 생활습관, 우울, 부부친밀도를 포괄적으로 포함하여 삶의 질에 미치는 영향을 확인하였다는 것에 의의가 있다.

## Conclusion

본 연구는 유방암 생존자의 삶의 질에 영향을 미치는 요인을 확인하여 삶의 질을 높이기 위해 필요한 간호중재 프로그램을 개발하기 위한 기초자료를 제공하고자 수행되었다. 연구 대상자의 삶의 질은 우울이 낮을수록, 부부친밀도가 높을수록, 생활습관이 좋을수록 삶의 질이 높아지는 것으로 나타났다.

이러한 연구를 토대로 유방암 생존자의 삶의 질을 높이기 위해서는 우울을 낮추고, 건강한 생활습관을 이행하고 유지할 수 있도록 하며 부부친밀도를 높일 수 있는 중재가 필요하다. 특히 유방암 여성에게 건강한 생활습관의 중요성에 대한 교육과 이행을 할 수 있도록 하고, 본 연구의 결과에서 확인된 영향 요인들을 고려한 효율적인 간호중재 프로그램의 개발뿐 아니라 그러한 프로그램의 제공을 통하여 효과를 확인하는 연구를 제언한다.

## Figures and Tables

**Table 1.** General characteristics (N=146)

Variable	Categories	n (%) or Mean ± SD
Age (year)	< 49	44 (30.1)
	50–59	55 (37.7)
	≥ 60	47 (32.2)
		55.2 ± 9.6
Spouse’s age (year)	< 49	30 (20.5)
	50–59	55 (37.7)
	≥ 60	61 (41.8)
		58.2 ± 11.5
Educational level	< High school	25 (17.1)
	High school	67 (45.9)
	≥ College	54 (37.0)
Occupation	Yes	55 (37.7)
	No	91 (62.3)
Religion	Yes	109 (74.7)
	No	37 (25.3)
Monthly income (× 10,000, KRW)	< 199	36 (24.6)
	200–399	65 (44.5)
	≥ 400	45 (30.9)
Cancer stage	1	19 (13.0)
	2	64 (43.8)
	3	34 (23.3)
	4	29 (19.9)
Time since diagnosis (year)	<1	61 (41.8)
	1–3	32 (21.9)
	3–5	22 (15.1)
	≥5	
Type of treatment	OP	38 (26.0)
	OP+Chemo	27 (18.5)
	OP+RT	16 (11.0)
	OP+Chemo+RT	60 (41.1)
	HT	5 (3.4)

Chemo: Chemotherapy; HT: hormone therapy; KRW: Korean won; OP: operation; RT: radiation therapy.

**Table 2.** Levels of lifestyle, depression, marital intimacy, and quality of life (N=146)

Variable	Categories	n (%) or Mean ± SD	Min–Max	Range
Lifestyle		141.13 ± 23.48	82–194	52–208
	Health responsibility	24.97 ± 5.08	14–35	9–36
	Physical activity	18.60 ± 5.62	8–31	8–32
	Nutrition	26.17 ± 4.57	14–36	9–36
	Spiritual growth	25.59 ± 5.61	11–36	9–36
	Interpersonal relations	26.72 ± 4.95	12–36	9–36
	Stress management	21.64 ± 4.19	10–31	8–32
Depression		6.52 ± 5.31	0–20	0–21
	Yes (≥ 10)	106 (72.6)		
	No (< 10)	40 (27.4)		
Marital intimacy		45.47 ± 9.95	31–68	15–75
	Perceptional	17.20 ± 4.27	10–25	5–25
	Emotional	16.09 ± 3.69	10–25	5–25
	Sexual	15.39 ± 4.10	8–19	5–25
Quality of life		86.98 ± 24.00	22–138	0–148
	Physical	17.41 ± 7.19	3–28	0–28
	Social/family	14.78 ± 5.14	3–28	0–28
	Emotional	14.89 ± 5.50	4–24	0–24
	Functional	17.71 ± 5.76	8–28	0–28
	Breast cancer-specific	22.17 ± 6.92	0–36	0–40

**Table 3.** Comparison of quality of life according to participants’ characteristics (N=146)

Variable	Categories	Quality of life	
		Mean ± SD	t, F (*p*)
Age (year)		86.98±24.00	7.25 (.001)
	< 49^a^	75.93 ± 23.92	a < b, c^†^
	50–59^b^	91.70 ± 22.26	
	≥ 60^c^	91.80 ± 23.07	
Spouse’s age (year)		86.98±24.00	5.57 (.005)
	< 49^a^	75.06 ± 25.22	a < b, c^†^
	50–59^b^	87.52 ± 23.57	
	≥ 60^c^	92.36 ± 22.01	
Educational level	< High school	91.92 ± 25.68	0.64 (.531)
	High school	86.10 ± 23.69	
	≥ College	85.79 ± 23.78	
Occupation	Yes	92.87 ± 23.75	2.34 (.021)
	No	83.42 ± 23.58	
Religion	Yes	89.90 ± 15.81	–0.35 (.730)
	No	84.83 ± 29.18	
Monthly income (×10,000, KRW)	< 199	86.52 ± 26.05	1.36 (.261)
	200–399	84.01 ± 24.68	
	≥ 400	91.64 ± 20.92	
Cancer stage	1	94.00 ± 23.96	2.66 (.050)
	2	91.03 ± 24.06	
	3	80.17 ± 23.17	
	4	81.44 ± 22.81	
Time since diagnosis (year)	<1	84.96 ± 22.78	0.95 (.420)
	1–3	86.28 ± 23.23	
	3–5	94.81 ± 28.06	
	≥5	86.12 ± 24.16	
Type of treatment	OP	89.55 ± 23.30	.50 (.733)
	OP+Chemo	84.22 ± 24.52	
	OP+RT	92.68 ± 25.64	
	OP+Chemo+RT	82.25 ± 24.88	
	HT	85.00 ± 8.21	

Chemo: Chemotherapy; HT: hormone therapy; KRW: Korean won; OP: operation; RT: radiation therapy.

† Scheffé test.

**Table 4.** Correlations among research variables (N=146)

Variable	r (*p*)			
	Lifestyle	Depression	Marital intimacy	Quality of life
Depression	–.24 (.004)	1		
Marital intimacy	.48 (< .001)	–.39 (< .001)	1	
Quality of life	.49 (< .001)	–.72 (< .001)	.45 (< .001)	1

**Table 5.** Influencing factors on quality of life (N=146)

Factor	Model 1			Model 2			Model 3			Model 4		
	B	β	t (*p*)	B	β	t (*p*)	B	β	t (*p*)	B	β	t (*p*)
(Constant)	95.5		14.09 (< .001)	48.03		3.82 (< .001)	72.62		7.86 (< .001)	61.90		6.52
Age	16.64	.33	2.81 (.005)	12.19	.24	2.16 (.032)	9.15	.18	3.26 (.035)	8.88	.12	2.27 (.051)
Spouse’s age	2.24	.04	0.34 (.744)	2.12	.04	0.34 (.737)	0.06	.01	0.01 (.989)	0.38	.01	0.09 (.930)
Occupation	–13.25	–.27	–3.44 (.001)	–10.45	–.21	–2.84 (.005)	–2.17	–.05	–0.80 (.428)	–1.08	–.02	–0.41 (.686)
Lifestyle				0.35	.09	4.39 (<.001)	0.22	.22	3.69 (<.001)	0.13	.13	2.17 (.031)
Depression							–2.85	–.65	–11.62 (< .001)	–2.78	–.63	–11.71 (< .001)
Marital intimacy										0.45	.19	3.32 (.001)
	R^2^ = .152, Adjusted R^2^ = .134			R^2^ = .254, Adjusted R^2^ = .233			R^2^ = .620, Adjusted R^2^ = .607			R^2^ = .648, Adjusted R^2^ = .633		
	F (*p*) = 8.49 (< .001)			F (*p*) = 12.01 (< .001)			F (*p*) = 45.74 (< .001)			F (*p*) = 42.67 (< .001)		

Dummy variable reference were age (<49 y), spouse's age (<49 y), and occupation (Yes).
